# Evaluation of alignment of the reimbursement medicines list for children in Albania with the WHO essential medicines list for children

**DOI:** 10.1080/20523211.2023.2290100

**Published:** 2023-12-16

**Authors:** E. Petro, V. Perumal-Pillay, A. K. Mantel-Teeuwisse, H. A. van den Ham, F. Suleman

**Affiliations:** a Local Healthcare Unit, Durres, Albania; b WHO Collaborating Centre for Pharmaceutical Policy and Evidence Based Practice, Discipline of Pharmaceutical Sciences, School of Health Sciences, University of KwaZulu-Natal, Durban, South Africa; c Utrecht WHO Collaborating Centre for Pharmaceutical Policy and Regulation, Division of Pharmacoepidemiology and Clinical Pharmacology, Utrecht Institute for Pharmaceutical Sciences (UIPS), Utrecht University, Utrecht, the Netherlands

**Keywords:** Essential medicine list for children, reimbursement list, access, medicines, children, Albania

## Abstract

**Background:**

The WHO Essential Medicine List for Children was released on the 30th anniversary of the general Essential Medicine List in 2007, to recognise special needs for medicines in children, and to promote the inclusion of paediatric medicines in national procurement programmes. This study aimed to investigate the alignment of the medicines included in the Albanian reimbursement medicines list of the Mandatory Healthcare Insurance Fund (AMHIF) and the Essential Medicine List for Children.

**Methods:**

A quantitative evaluation was performed to compare the paediatric medicines included in the 2022 list of the AMHIF and the 2021 WHO Essential Medicine List for Children. In addition, vaccines in the Albanian vaccination programmes for children were compared to the ones listed on the WHO Essential Medicine List for Children.

**Results:**

Both lists had a total of 284 active ingredients in common, whereas 14 of 24 vaccines were found to be in common in the Essential Medicine List for Children list and the Albanian vaccination programmes.

**Conclusions:**

This is the first study in Albania to investigate the alignment of the WHO EMLc and AMHIF list. In case of the same active ingredient there were many deviations in terms of dosage form, strength and indication.

## Introduction

Access to health care and essential medicines is increasingly being viewed as a fundamental human right (Hogerzeil, 2003). Medicines are critical to health systems strengthening; without medicines, confidence in the local health system declines (Leach et al., 2014). The World Health Organization (WHO) has previously estimated that one-third of the world’s population lacked access to essential medicines (World Health Organization, 2004). Essential medicines are those that satisfy the priority health needs of the population (United Nations, 2022). They ‘are intended to be available within the context of functioning health systems at all times in adequate amounts, in the appropriate dosage forms, with assured quality, and at a price the individual and the community can afford’ (World Health Organization, 2022). Medicines cannot have a positive impact on health unless they are used appropriately (Jimmy and Jose, 2011). Essential medicines are one of the core components to promoting rational usage and prescription of medicines (Kar et al., 2010). However, there is still a global paucity of age-appropriate formulations of paediatric medicines to treat and prevent a variety of conditions, especially in low- and middle-income countries (LMICs) (Richey et al., 2017; Ainscough et al., 2018; Orubu and Tuleu, 2017; Zahn et al., 2020; Best et al., 2011). Research efforts have traditionally focused on measuring access to medicines for the general population, without particular consideration for medicines for children. As a result, there is a major gap in our understanding of accessibility of medicines for children (Joosse et al., 2022).

Children differ from adults in many aspects of pharmacotherapy, including capabilities for medicine administration, medicine-related toxicity and taste preferences (Ivanovksa et al., 2014). It is essential that paediatric medicines are formulated to best suit a child’s age, size, physiological condition and treatment requirements. To ensure adequate treatment of all children, different routes of administration, dosage forms and strengths may be required (Ivanovksa et al., 2014). Yet, children have been commonly considered ‘therapeutic orphans’ because the majority of medicines on the market have not been studied or authorised for use in the paediatric (sub)population (Shirkey, 1968). As a global action to improve access to child-specific medicines, the WHO Essential Medicines List for Children (EMLc) was released on the 30th anniversary of the general EML in 2007. The aim of the EMLc is to recognise special needs for medicines in children, and to promote the inclusion of paediatric medicines in national procurement programmes (World Health Organization, 2007). Access to affordable, quality-assured essential medicines for children is crucial to reducing the financial burden of care to parents and guardians and improving population health worldwide (Ozawa et al., 2019). To improve access to medicines for children, it is necessary to evaluate the accessibility of medicines for children. The availability of medicines for children in both public sectors and private sectors in low-income and middle-income countries is low (Chen et al., 2021).

Very little is known about the selection process of paediatric medicines authorised and marketed in Albania that are included in the AMHIF list. Albania has not yet developed a National Essential Medicine List for children guided by the WHO EMLc, nor are there any indications that the WHO EMLc is considered when the paediatric medicines in the AMHIF list are updated annually. The aim of this study was to investigate the alignment of the active ingredients on the WHO EMLc (2021) and the active ingredients included in the 2022 Albanian Reimbursement medicines List of AMHIF. The WHO EMLc guides the rational selection and use of medicines in children aged 0–12 years (Braine, 2007). It is crucial to ensure the availability of proper formulations for children in order to avoid administration errors, adverse drug reactions and poor therapeutic outcomes in the paediatric population. To have a complete comparative analysis of all the categories included in the WHO EMLc we additionally investigated the alignment of the vaccines in the WHO EMLc with the vaccines included in the National Immunization Programme and the Seasonal Influenza Immunization Programme.

Albania is an upper-middle-income country located in south-eastern Europe. The population of Albania on 1 January 2021, was almost 3 million inhabitants (Instituti i Statistikave, Republika e Shqiperise, 2022). The healthcare system in Albania is mostly public, and organised at the primary, secondary, and tertiary service levels. Approximately 413 public healthcare clinics offer primary and secondary healthcare services, and 42 public hospitals offer tertiary healthcare services. Pharmaceutical and dental services are almost entirely private (Jacellari, 2019). The Albanian legal framework lacks regulation on medicinal products for paediatric use, compared to the European Union, creating a gap in the level of knowledge regarding the access to paediatric medicines and their rational usage (Roshi et al., 2023).

The Albanian Mandatory Health Insurance Fund (AMHIF) was established in 1995 as a single purchasing agency and includes services provided by a combination of public and contracted private primary-care centres, hospitals and contracted providers of medicines, medical products, and other treatment. The Government transfers funds to the AMHIF to cover people who are economically inactive, such as children aged under 18 years, students under 25 years, pensioners, people registered to receive social assistance or disability benefits, registered unemployed people, asylum seekers and a few other categories set out in special laws (Tomini & Tomini, 2020). The list of covered outpatient medicines is formulated by the AMHIF and approved by governmental decree. The reimbursement list is reviewed on an annual basis. Inpatient care in public facilities with referral is free of charge for people covered by the AMHIF. The pharmacies contracted with AMHIF reimburse the medicines from the reimbursement list (Fondi i Sigurimit te Detyrueshem te Kujdesit Shendetesor, 2022). The list has expanded over time, increasing from 278 medicines in 1996 to 540 in 2018 (Tomini & Tomini, 2020).

## Method

### Data sources

The 8th edition of the WHO EMLc published in September 2021 (WHO 2021) and the AMHIF list published in 2022 (Fondi i Sigurimit te Detyrueshem te Kujdesit Shendetesor, 2022) were converted into Microsoft Excel workbooks, in order to make the comparison between active ingredients easier. Both lists follow the International Non-proprietary Names (INN, generic names) designation for medicines (World Health Organization, 2014). The WHO EMLc list is organised into 30 therapeutic categories according to pharmacological classes whilst the AMHIF list is organised by the Anatomical Therapeutic Chemical (ATC) classification into 14 groups. The WHO EMLc is further differentiated into core and complementary lists. The core list presents a list of minimum medicine needs for a basic health-care system, listing the most efficacious, safe and cost-effective medicines for priority conditions. The complementary list presents essential medicines for priority diseases, for which specialised diagnostic or monitoring facilities, and/or specialist medical care, and/or specialist training are needed (WHO, 2021). Both the core and complementary lists were included in the comparison.

### Data extraction and comparison between the WHO EMLc list and AMHIF list

An observational descriptive study was performed to quantitatively analyse the active ingredients included in both lists. The WHO EMLc list was compared to the AMHIF list for the total number of active ingredients that was similar in both, including where there was duplication in the lists for each active ingredient. Duplicates were defined as active ingredients which were appearing more than once in each list (e.g. morphine appearing in the EMLc in Anaesthetics, preoperative medicines and medical gases category as well as in Medicines for pain and palliative care category) and were counted once. The active ingredients listed in the WHO EMLc with the square box symbol (□) are intended to indicate therapeutic alternatives to that category of active ingredients. Thus, if therapeutic alternatives exist in the AMHIF list, these were considered to be aligned to the WHO EMLc. An assessment was performed for the active ingredients of the AMHIF list alone, identifying the total number of molecules, the duplicates and unique molecules for each ATC category.

Active ingredients of the WHO EMLc list were compared with the active ingredients on the AMHIF List to identify (1) the active ingredients found on both lists, and (2) their alignment accordingly to dose form, strength and listed indication, for which matching codes were assigned (see Supplementary Table 1).

The alignment was examined by means of the categories listed below:

- Duplicates
- No difference in the active ingredients in terms of dose form, dosage and listed indication (ND).
- Just one difference in the active ingredient-amongst which we observed three categories: active ingredients with different dosage (DD), active ingredients with different dosage form (DF) and active ingredients with different listed indications (DI).
- Two differences in the active ingredients amongst which we observed three categories: active ingredients with different dosage and different dose formulation (DDF), active ingredients with different dosage and different listed indication (DDI) and active ingredient with different dose formulation and different indication (DFI).
- All aspects (dose, formulation and indication) different for the active ingredients (DDFI)
- Therapeutic alternative (square box in WHO EMLc)

Active ingredients deleted from the WHO EMLc (2021) were checked against the AMHIF list to identify if they were present at all in the latter. Active ingredients that appeared only in the AMHIF were counted as well.

Comparison between the category of Immunologicals and Vaccines of the WHO EMLc and the National Immunization Programme and the Seasonal Influenza Immunization Programme in Albania were undertaken as there are no vaccines included in the AMHIF list. The National Immunization Programme is obligatory for children 0–18 years old and is offered in the primary healthcare centres. At the same time each year the Seasonal Influenza Immunization Programme is implemented according to priority groups, children included.

## Results

### Overall comparison

The 8th edition of the WHO Model EMLc had in total 471 active ingredients (including duplicates), of which 336 active ingredients were found in the core list and 135 active ingredients in the complementary list. For each category of the WHO EMLc the total number of active ingredients missing on the AMHIF list was identified (see Supplementary Table 2). The AMHIF list published in 2022 had in total of 1166 active ingredients (including duplicates) from which 620 are unique active ingredients and 239 duplicates as it is shown in Supplementary Table 3.

Overall, there were a total of 284 active ingredients (including different dosage forms and strengths) common to both lists, with 34 therapeutic alternatives (square box medicines) appearing in the WHO EMLc also present in the AMHIF list. Among these, 137 active ingredients (48.2%) in the AMHIF list had no difference in any aspects (dosage form, strength and indication) with those on the WHO EMLc. There were 98 active ingredients (34.5%) that differed just in one aspect in the AMHIF list compared to its counterparts in the WHO EMLc, from which 54 active ingredients (19%) had a different dosage, 43 active ingredients (15.1%) had a different dose formulation and 1 active ingredient (0.35%) had a different listed indication. Further 49 active ingredients (17.3%) had differences in more than one characteristic (formulation, dosage and/or indication) compared to the WHO EMLc. From these, only 10 active ingredients (3.52%) differed in all the characteristics dose form, dosage and listed indication whereas 31 active ingredients (10.9%) differed in the dose formulation and dosage and 8 active ingredients (2.81%) differed in dose formulation and listed indication (see Table 1). In terms of the categories of the WHO EMLc that were mostly aligned to the AMHIF list were the active ingredients for mental and behavioural disorders (3/3), the cardiovascular category (3/4), active ingredients for disease of joints (2/3) and the antimigraine category (2/3), excluding duplicates. In contrast, active ingredients in the categories of blood products of human origin and plasma substitutes, antiseptics and disinfectants, the peritoneal dialysis solution and dental preparations of the WHO EMLc were not found in the AMHIF.

**Table 1.** Level of alignment of the active ingredients found in both lists.

EMLc_Category	Level of alignment							
	ND (*N* = 137)	DD (*N* = 54)	DF (*N* = 43)	DI (*N* = 1)	DDFI (*N* = 10)	DDF (*N* = 31)	DFI (*N* = 8)	Total** (*N* = 284)
Anaesthetics, preoperative medicines and medical gases	2		1			3		6
Medicines for pain and palliative care	7	6	9					22
Antiallergics and medicines used in anaphylaxis	5		1			4		10
Antidotes and other substances used in poisonings	1							1
Anticonvulsants/Antiepileptics	9	1	3		4	1	2	20
Anti-infective medicines	35	16	11			8	2	72
Antimigraine medicines			4			1		5
Immunomodulators and antineoplastics	14	6	6			1		27
Medicines affecting the blood	17	6				3		26
Blood products of human origin and plasma substitutes								
Cardiovascular medicines	3	2						5
Dermatological medicines (topical)	3	4				1		8
Diagnostic agents	1							1
Antiseptics and disinfectants								
Diuretics	4	2						6
Gastrointestinal medicines	9	1	1					11
Medicines for endocrine disorders	9	3	3			4		19
Muscle relaxants and cholinesterase inhibitors			1					1
Ophthalmological preparations	3		1					4
Medicines for reproductive health and perinatal care					4			4
Peritoneal dialysis solution								
Medicines for mental and behavioural disorders	5	1	1					7
Medicines acting on the respiratory tract	4	5				2	2	13
Solutions correcting water, electrolyte and acid-base disturbances	4							4
Vitamins and minerals		1	1			3		5
Ear, throat and nose medicines	1				2			3
Medicines for joint disorders	1			1			2	4
Dental preparations								

Note: ND-No Difference in terms of Dose Form,Strength & Indication; DD-Different Dosage; DF-Different Dose Form; DI-Different Indication; DDFI-Different Dosage & Different Dose Form & Different Indication; DDF-Different Dosage & Different Dose Form; DFI-Different Dose Form & Different Indication.

*In the table the information for matching code 10- DDI-Different Dosage & Different Indication is excluded due to no active ingredient falling in this category.

**The Total column in the table includes duplicates.

### Comparison by categories in the AMHIF and WHO EMLc

Active ingredients found on both the WHO EMLc list and AMHIF sorted by ATC categories (see Table 2) are 228 unique active ingredients, with 101 duplicates (this is in total 443 active ingredients as duplicates appeared more than once).

**Table 2.** Level of alignment of ATC categories in the reimbursement list of AMHIF with the WHO EMLc.

ATC categories	Active ingredients found on both AMHIF and EMLc			Level of alignment of ATC categories with WHO EMLc						
	Total active ingredients (*N* = 443)	Total Unique active ingredients (*N* = 228)	Total duplicates**(*N* = 101)	ND (*N* = 232)	DD (*N* = 102)	DF (*N* = 52)	DI (*N* = 3)	DDFI (*N* = 7)	DDF (*N* = 42)	DFI (*N* = 5)
Alimentary tract and metabolism	57	33	9	31	3	9			14	
Blood and blood forming organs	35	20	10	15	7		3		7	3
Cardiovascular system	22	8	5	12	10					
Dermatologicals	17	8	5	7	6				4	
Genito-urinary system	1	1								1
Systemic hormonal preparations	21	11	4	15	4	2				
Antiinfectives for systemic use	110	50	26	44	50	10			6	
Antineoplastic and immunomodulating agents	33	19	6	25	3	4			1	
Musculo-skeletal system	15	6	4			12		1	2	
Nervous system	61	37	13	29	10	12		6	4	
Antiparasitic products	3	3		1		1			1	
Respiratory system	38	17	11	26	8				3	1
Sensory organs	9	4	4	7		2				
Various	21	11	4	20	1					

Note: ND-No Difference in terms of Dose Form,Strength & Indication; DD-Different Dosage; DF-Different Dose Form; DI-Different Indication; DDFI-Different Dosage & Different Dose Form & Different Indication; DDF-Different Dosage & Different Dose Form; DFI-Different Dose Form & Different Indication* In the table is not displayed the information for matching code 10- DDI-Different Dosage & Different Indication (not found any active ingredient falling in this category) **Duplicate active ingredients can appear more than once sometimes in the list, however they are counted only once.

Among the 443 active ingredients with duplicates that were found, 232 active ingredients (52.4%, duplicates included) were fully aligned with the EMLc list with no difference in terms of dose form, strength and indication. There were 157 active ingredients (35.4% duplicates included) that differed just in one aspect in the AMHIF list compared to its counterparts in the WHO EMLc, from which 102 active ingredients (23%) had a different dosage, 52 active ingredients (11.7%) had a different dose formulation and 3 active ingredients (0.67%) had a different listed indication. Further 54 active ingredients (12.2%, duplicates included) had differences in more than one characteristic (formulation, dosage and/or indication) compared to the WHO EMLc. From these 7 active ingredients differed in all the characteristics dose form, dosage and listed indication whereas 42 active ingredients differed just in the dose formulation and dosage and 5 active ingredients differed just in dose formulation and listed indication.

The AMHIF list had 723 active ingredients in total, duplicates included, that were not present in the WHO EMLc, among which 392 were unique active ingredients. The active ingredients present only in the AMHIF list were mostly found in the cardiovascular system (*n* = 271, duplicates included) nervous system (*n* = 103, duplicates included) and antineoplastic and immunomodulating agents (*n* = 70, duplicates included) category.

### Deleted active ingredients

We found that none of the active ingredients, that were deleted from the 2021 WHO EMLc list, were present in the AMHIF list, as it is shown in Supplementary Table 4.

### Comparison of immunologicals and vaccines

In total there were 24 vaccines under the category of Immunologicals and Vaccines in the WHO EMLc list, of which 10 vaccines were not present in the Albanian National Immunization Programme. These included Japanese encephalitis vaccine, tick borne encephalitis vaccine, yellow fever vaccine, cholera vaccine, dengue vaccine, hepatitis A vaccine, meningococcal meningitis vaccine, rabies vaccine, typhoid vaccine and varicella vaccine. Among the 14 vaccines that were present, 13 were included in the Albanian National Immunization Programme and one, the influenza vaccine, was applicable to priority groups (children included) and thus recommended by the National Institute of Public Health (see Table 3).

**Table 3.** Comparison of the immunologicals and vaccines.

Vaccines in EMLc	Vaccines in the Albanian National Immunization Programme									
	Age/years old									
	1	2	5	6	13	18–19				
	Birth	2 months	4 months	6 months	12 months					
BCG vaccine	BCG									
Diphtheria vaccine		DTP-HepB_Hib	DTP-HepB_Hib	DTP-HepB_Hib		DTP		DT	Td	Td
Haemophilus influenza type b vaccine		DTP-HepB_Hib	DTP-HepB_Hib	DTP-HepB_Hib						
Hepatitis B vaccine	HepB									
Measles vaccine					MMR		MMR			
Pertussis vaccine		DTP-HepB_Hib	DTP-HepB_Hib	DTP-HepB_Hib		DTP				
Pneumococcal vaccine		PCV	PCV		PCV					
Poliomyelitis vaccine		IPV	IPV	bOPV		IPV/bOPV		IPV/bOPV		
Rotavirus vaccine		Rota	Rota	Rota						
Rubella vaccine					MMR		MMR			
Tetanus vaccine		DTP-HepB_Hib	DTP-HepB_Hib	DTP-HepB_Hib		DTP		DT	Td	Td
Mumps vaccine					MMR		MMR			
Human papilloma virus (HPV) vaccine									HPV	
Influenza Vax	**Seasonal Influenza Immunization Program (accordingly to priority groups)**									

Note: BCG-Tuberculosis vaccine; HepB-Hepatitis B Viral vaccine; DTP-HepB_Hib- Diphteria, Tetanus, Pertusis, HepatitisB, Haemophilus Influenza B Type vaccine; DTP-Diphteria, Tetanus, Pertusis vaccine; DT-Diphteria, Tetanus vaccine; Td-Diphteria, Tetanus vaccine; MMR-Measles, Mumps, Rubella vaccine; PCV-Pneumococcal vaccine; IPV- Inactivated poliomyelitis vaccine; bOPV – Bivalent oral poliomyelitis vaccine; Rota- Rotavirus vaccine; HPV- Human Papilloma Virus vaccine.

## Discussion

This study showed that the Albanian reimbursement list and the WHO EMLc had the most active ingredients in common. Some but not all of the medicines that were not included in the AMHIF list were covered through other channels. The Albanian National Immunization Programme covered approximately 60% of the vaccines listed on the WHO EMLc.

There is no other similar study in Albania, and even fewer globally looking specially at alignment of country EML and WHO EMLc. A study was conducted in Serbia that investigated the availability of paediatric-evaluated formulations in 2017. The WHO EMLc list was used as a comparison tool and it was found that 51% of the medicines in the WHO EMLc were available in Serbia (Božić et al., 2017). In 2015, a study by Kazaryan and Vardanyan was conducted in the Republic of Armenia (RA) to examine the access to essential medicines for children. The Armenian Essential Medicines List of 2007 and 2013 as well as the Lists of medicines registered in Armenia (2011-2013) were analysed. The results showed that in 2013, 57.7% of the medicines from WHO EMLc were included on the National List of Essential Medicines of RA (Kazaryan and Vardanyan, 2015). In our study, 71% of the WHO EMLc active ingredients found to be in common were aligned to the AMHIF list.

A research paper published in 2016 addressed the lack of available paediatric medicines in Albania for two groups of selected medicines, H_2_-antihistaminics and proton pump inhibitors, and showed that these medicines may not be age-appropriate; in spite of the fact that they are authorised for such use (Dosti and Malaj, 2016). This study found that across all the categories 92/284 active ingredients in the AMHIF list had different formulation than the active ingredients in the WHO EMLc, indicating that there are still concerns regarding the lack of age-appropriate active ingredients in the AMHIF list.

The findings of this study showed that the active ingredients in the categories of blood products of human origin and plasma substitutes, antiseptics and disinfectants, the peritoneal dialysis solution and dental preparations of the WHO EMLc were not found in the AMHIF List. The blood products of human origin and plasma substitutes are managed and provided to the hospitals for the patients’ needs through the National Center of Blood Transfusion, and all the prevention and treatment of dental problems are managed in the Dental Services of the Local Healthcare Units for children 0–18 years old without any fee. Thus, the medicines for these categories are accessible to the population through other channels. Further investigation highlighting the reasons for exclusion of antiseptics and disinfectants and the peritoneal dialysis solution from the AMHIF list needs to be conducted.

In a study conducted by Perumal-Pillay and Suleman (2016) looking at South Africa’s Standard Treatment Guidelines/Essential Medicine List (Primary Health Care and Adult Hospital 2012, and Paediatric Hospital 2013) in comparison with the WHO EMLs (18th list, 4th list for children) and 2012 priority life-saving medicines for women and children (PMWC) list found that the lists were somewhat aligned for most conditions as only 3 of 14 medicines and 11 of 20 vaccines were absent from SA STG/EMLs. In South Africa the immunization schedule is as per the Expanded Programme on Immunization (EPI-SA), which does include the Rotavirus vaccine which is not listed in the SA STG/EMLs (PHC 2008 and Paediatric 2013), though reference to the EPI-SA is made in the STG/EMLs. Vaccines such as Rubella and Mumps as part of the Measles/Mumps/Rubella (MMR) vaccine, HPV, Meningococcal and Hepatitis A are present on the PMWC list, but not listed on the EPI-SA and are only available in the private sector healthcare (Perumal-Pillay and Suleman, 2016). In our study, we found that in the Albanian National Immunization Program and the Seasonal Influenza Immunization Program there were 10 out of 24 vaccines absent when compared to the WHO EMLc, and there was no epidemiological relevance for the missing vaccines.

It is important to highlight the fact that in order to have complete information on the accessibility of essential medicines for children research should be conducted not only in the national reimbursement list of medicines, but in any other national programme approved by the national authorities such as the National Immunization Programme and the Seasonal Influenza Immunization Programme in the case of Albania. The Institute of Public Health has approved the programmes and from time to time the National Immunization Programme has expanded in order to completely align with the WHO recommended vaccine for children in all countries. So, from the 1st of October 2019 the Rotavirus vaccine was added and lastly from October 2022 the HPV vaccine was added to the national immunization programme. There is no epidemiological relevance in our country for the 10 vaccines missing from the WHO EMLc list such as Japanese encephalitis vaccine, tick borne encephalitis, yellow fever vaccine, etc.

More studies looked at alignment of WHO EML to national EMLs. A comparative analysis of medicines included in the national essential medicines lists with the WHO EML in 137 countries was performed by Persaud et al. (2019). The findings of the study showed that each national list contained between 44 and 983 medicines (Persaud et al., 2019) from WHO EML. Even though we looked at the alignment of the WHO EMLc this fits in with our study that showed that overall, a total 284 medicines (with duplicates) were found both in the WHO EMLc and AMHIF list.

The alignment of the WHO EML with the national reimbursement list of medicines was studied in Croatia and the Federation of Bosnia and Hercegovina (Kadic et al., 2014; Mahmić-Kaknjo and Marušić 2015). The WHO Essential Medicines List was used as a tool to assess the appropriateness of Insurance Coverage Decisions in the Croatian National Medicine Reimbursement List in 2014. From the 2012 Croatian Institute of Health Insurance Basic list (CIHI list) 9 ATC categories with the highest expenditures were compared with the 2011 WHO EML. The result of the study showed that, of the 9 ATC categories compared, the lists shared 188 medicines (52.4% of the WHO EML and 36.9% of the CIHI list) (Kadic et al., 2014). A study conducted in 2015 in Bosnia and Hercegovina compared the Basic Medicines Lists of the Federation of Bosnia and Hercegovina (FBH Basic Lists (FBLs)) with the WHO Essential Medicines list and the evidence supporting the inclusion of additional medicines on the FBLs. The medicines form 9 ATC classes on the FBH list were compared with the 2013 WHO EML (core and complementary). There were 124 (46% of the WHO EML and 37% of the FBLs) medicines present in both lists, from which 27% had the same dose, formulation and indication in both lists (Mahmić-Kaknjo & Marušić, 2015). The results of our study found more active ingredients (284 active ingredients (with duplicates)) to be in common (71% of the WHO EMLc) which appeared as 443 active ingredients (with duplicates) through 14 ATC categories (38% of the AMHIF list). We however, looked at more ATC categories than the others which were limited to specific ATC categories. Therefore, is difficult to compare our study to the above-mentioned studies in order to reach a conclusion of the results.

The results of the studies highlight the need of using a quality evidence tool such as the WHO EML and EMLc when deciding on the inclusion or deletion of medicines from the national reimbursement lists. However, countries may have justification in excluding medicines from their lists based on epidemiological profile, budget constraints and registration status of medicines (Bigdeli et al., 2023).

## Strengths and limitations of the study

This is the first study that investigated the presence of the active ingredients of the WHO EMLc in the AMHIF list which is of great importance in terms of the accessibility to the population of essential medicines for children, since there is no national EMLc list developed yet. An important difference with similar studies looking at alignment is that we not only looked at the level of inclusion of active ingredients, but also at dosages, formulations and indications.

This study did not consider the specific disease burden of Albania while analysing the medicines present in the WHO EMLc. At the same time, the country’s GDP and the budget for medicines were not considered. The annual process of medicines selection for the AMHIF was not analysed in terms of the criteria of medicine’s addition or deletion from the list.

## Conclusion

This is the first study in Albania to investigate the alignment of the WHO EMLc and AMHIF list. The results provide insight as to the areas in which there are similarities in both lists in ensuring access to medicines for children in Albania, as recommended by the WHO EMLc. However, in case of the same ingredient there are many deviations in terms of dosage form, strength and indication, and that many child medicines listed on the WHO EMLc have not been included in the Albanian list, which both warrant further research.

## Abbreviations

ATC categories : Anatomical Therapeutical Chemical categories
AMHIF: Albanian Mandatory Health Insurance Fund
CIHI: Croatian Institute for Health Insurance
EML: Essential Medicine List
EMLc: Essential Medicine List for children
EPI: Expanded programme on immunization
FBH: Federation of Bosnia and Hercegovina
FBLs: FBH Basic List
PHC: Primary health care
PMWC: Priority life-saving medicines for woman and children
RA: Republic of Armenia
SA: South Africa
STG: Standard Treatment Guidelines
WHO: World Health Organization

## Supplementary Material

Supplemental Material

## Data Availability

All data generated or analysed during this study are included in this published article (and its supplementary information files).
