# Supplementary material for: Evaluation of alignment of the reimbursement medicines list for children in Albania with the WHO essential medicines list for children
Source: J Pharm Policy Pract. 2023 Dec 16;17(1):2290100. doi: 10.1080/20523211.2023.2290100 (PMC10775712; doi:10.1080/20523211.2023.2290100)
Supplement: Supplemental Material [file JPPP_A_2290100_SM9506.docx]

**Supplementary Table 1**: Matching codes in the alignment analysis

| **Code Number** | **Code Meaning** |
| --- | --- |
| 1 | Duplicates inside the EMLc |
| 2 | Active ingredients with different dosage (DD) |
| 3 | Active ingredients with no difference in terms of dosage, dosage form and indication (ND) |
| 4 | Different dosage and dose form active ingredients (DDF) |
| 5 | Different indication active ingredients (DI) |
| 6 | Active ingredients therapeutic alternative in the EMLc |
| 7 | Different dosage, dose form and indication active ingredients (DDFI) |
| 8 | Different dose form active ingredients (DF) |
| 9 | Different dose form and indication active ingredients (DFI) |
| 10 | Different dosage and different indication (DDI) |

**Supplementary Table 2**: Number of active ingredients in the WHO EMLc 2021 [n= 471 including duplicates]

| **Category** | **WHO EMLc_2021** | | | |
| --- | --- | --- | --- | --- |
|  | **Core list with duplicates (N=336)** | **Complementary list with duplicates (N=135)** | **Total active ingredients with duplicates**  **(N=471)** | **Total active ingredients with duplicates missing in the AMHIF**  **(N=335)** |
| Anaesthetics, preoperative medicines and medical gases | 14 |  | 14 | 12 |
| Medicines for pain and palliative care | 19 | 1 | 20 | 11 |
| Antiallergics and medicines used in anaphylaxis | 8 |  | 8 | 3 |
| Antidotes and other substances used in poisonings | 5 | 5 | 10 | 9 |
| Anticonvulsants/Antiepileptics | 10 | 2 | 12 | 5 |
| Anti-infective medicines | 116 | 34 | 150 | 121 |
| Antimigraine medicines | 3 |  | 3 | 1 |
| Immunomodulators and antineoplastics |  | 49 | 49 | 33 |
| Medicines affecting the blood | 7 | 10 | 17 | 8 |
| Blood products of human origin and plasma substitutes | 8 | 3 | 11 | 11 |
| Cardiovascular medicines | 3 | 1 | 4 | 1 |
| Dermatological medicines topical | 20 |  | 20 | 16 |
| Diagnostic agents | 4 | 1 | 5 | 4 |
| Antiseptics and disinfectants | 9 |  | 9 | 9 |
| Diuretics | 1 | 5 | 6 | 3 |
| Gastrointestinal medicines | 12 | 2 | 14 | 10 |
| Medicines for endocrine disorders | 10 | 7 | 17 | 12 |
| Immunologicals and vaccines | 29 |  | 29 | 15 |
| Muscle relaxants and cholinesterase inhibitors | 3 | 1 | 4 | 3 |
| Ophthalmological preparations | 18 | 1 | 19 | 16 |
| Medicines for reproductive health and perinatal care | 2 | 5 | 7 | 5 |
| Peritoneal dialysis solution |  | 1 | 1 | 1 |
| Medicines for mental and behavioural disorders |  | 3 | 3 |  |
| Medicines acting on the respiratory tract | 9 |  | 9 | 5 |
| Solutions correcting water, electrolyte and acid-base disturbances | 9 |  | 9 | 7 |
| Vitamins and minerals | 9 | 1 | 10 | 6 |
| Ear, throat and nose medicines | 5 |  | 5 | 4 |
| Medicines for joint disorders |  | 3 | 3 | 1 |
| Dental preparations | 3 |  | 3 | 3 |

**Supplementary Table 3**: Number of active ingredients in the AMHIF 2022 [n= 1166 including duplicates]

| **ATC categories** | **AMHIF_2022** | | |
| --- | --- | --- | --- |
|  | **Total active ingredients (N=1166)** | **Total Unique active ingredients (N=620)** | **Total duplicates* *(N=239)** |
| Alimentary tract and metabolism | 85 | 53 | 15 |
| Blood and blood forming organs | 68 | 39 | 13 |
| Cardiovascular system | 293 | 126 | 59 |
| Dermatological | 24 | 13 | 7 |
| Genito-urinary system | 46 | 30 | 8 |
| Systemic hormonal preparations | 35 | 21 | 8 |
| Anti-infective for systemic use | 132 | 61 | 30 |
| Antineoplastic and immunomodulating agents | 103 | 67 | 16 |
| Musculo-skeletal system | 51 | 29 | 12 |
| Nervous system | 164 | 100 | 34 |
| Antiparasitic products | 3 | 3 |  |
| Respiratory system | 85 | 42 | 19 |
| Sensory organs | 48 | 20 | 13 |
| Various | 29 | 16 | 5 |

**Duplicate active ingredients can appear more than once sometimes in the list, however they are counted only once

**Supplementary Table 4:** Alignment of medicines/formulations deleted from WHO EMLc 2021 in AMHIF list 2022

| **WHO EMLc-2021_Medicines/Formulations deleted** | | | **AMHIF_2022** |
| --- | --- | --- | --- |
| **Medicine** | **Dosage form** | **Dosage strength** | **Medicine same dosage form and strength recommendation** |
| Abacavir | dispersible tablet | 60 mg | Not present |
| Amikacin (section 6.2.5 antituberculosis medicines only) | powder for injection | 100 mg | Not present |
|  |  | 500 mg | Not present |
|  |  | 1 g | Not present |
| Amoxicillin + clavulanic acid (Section 6.2.5 Antituberculosis medicines only) | oral liquid | 125 mg+ 31,25 mg/ml | Not present |
| Atazanavir | solid oral dosage form | 100 mg | Not present |
| Efavirenz | tablet (scored) | 200 mg | Not present |
| Isoniazid | tablet (scored) | 50 mg | Not present |
| Lamivudine | tablet | 150 mg | Not present |
| Lamivudine+nevirapine+zidovudine | tablet | 30 mg+50 mg+60 mg | Not present |
| Linezolid (Section 6.2.5 Antituberculosis medicines only) | Injection for IV administration | 2 mg/ml in 300 ml bag | Not present |
|  | tablet | 400 mg | Not present |
| Lopinavir + ritonavir | oral liquid | 400 mg+ 100 mg/5 ml | Not present |
| Oseltamivir | oral powder | 12 mg/ml | Not present |
| p-aminosalicylic acid | tablet | 500 mg | Not present |
| Pyrazinamide | tablet (scored) | 150 mg | Not present |
| Raltegravir | tablet (chewable) | 100 mg | Not present |
|  | tablet | 400 mg | Not present |
| Ritonavir | oral liquid | 400 mg/5 ml | Not present |
|  | oral powder | 100 mg in sachet | Not present |
